# Efficacy of heterologous boosting against SARS-CoV-2 using a recombinant interferon-armed fusion protein vaccine (V-01): a randomized, double-blind and placebo-controlled phase III trial

**DOI:** 10.1080/22221751.2022.2088406

**Published:** 2022-08-01

**Authors:** Xuan-Yi Wang, Syed Faisal Mahmood, Fang Jin, Wee Kooi Cheah, Muhammad Ahmad, Mian Amjad Sohail, Waheed Ahmad, Vijaya K. Suppan, Muneeba Ahsan Sayeed, Shobha Luxmi, Aik-Howe Teo, Li Yuan Lee, Yang-Yang Qi, Rong-Juan Pei, Wei Deng, Zhong-Hui Xu, Jia-Ming Yang, Yan Zhang, Wu-Xiang Guan, Xiong Yu

**Affiliations:** a Shanghai Institute of Infectious Disease and Biosecurity, Fudan University, Shanghai, People’s Republic of China; b Key Laboratory of Medical Molecular Virology of MoE & MoH, and Institutes of Biomedical Sciences, Fudan University, Shanghai, People’s Republic of China; c Children’s Hospital, Fudan University, Shanghai, People’s Republic of China; d Hospital of Aga Khan University, Karachi, Pakistan; e State Key Laboratory of Respiratory Disease, National Clinical Research Center for Respiratory Disease, Guangzhou Institute of Respiratory Disease, Guangzhou, People’s Republic of China; f Guangzhou Joincare Respiratory Medicine Co., Ltd, Guangzhou, People’s Republic of China; g Department of Medicine and Clinical Research Centre, Taiping Hospital, Perak, Malaysia; h Pulmonology & Critical care, Central Park Teaching Hospital, Lahore, Pakistan; i Shifa International Hospitals Ltd, Islamabad, Pakistan; j Avicenna Medical College, Lahore, Pakistan; k Clinical Research Center, Sultan Abdul Halim Hospital, Kedah, Malaysia; l Department of Infectious Diseases, Sindh Infectious Diseases Hospital and Research Centre, Dow University of Health Sciences, Karachi, Pakistan; m Dow University of Health Sciences, Karachi, Pakistan; n Penang General Hospital and Info Kinetics Clinical Research Centre, Pulau Pinang, Malaysia; o CRC Seri Manjung Hospital, Perak, Malaysia; p Wuhan Institute of Virology, Chinese Academy of Sciences, Wuhan, People’s Republic of China; q Livzon Mabpharm Inc., Zhuhai, People’s Republic of China; r Joincare Pharmaceutical Group Industry Co., Ltd., Shenzhen, People’s Republic of China

**Keywords:** Efficacy, heterologous boosting, subunit vaccine, clinical trial, fusion protein

## Abstract

Waning of neutralizing titres along with decline of protection efficacy after the second dose of COVID-19 vaccines was observed, including China-made inactivated vaccines. Efficacy of a heterologous boosting using one dose of a recombinant SARS-CoV-2 fusion protein vaccine (V-01) in inactivated vaccine-primed population was studied, aimed to restore the immunity. A randomized, double-blind and placebo-controlled phase III trial was conducted in healthy people aged 18 years or older in Pakistan and Malaysia. Each eligible participant received one dose of the V-01 vaccine developed by Livzon Mabpharm Inc. or placebo within the 3-6 months after the two-dose primary regimen, and was monitored for safety and efficacy. The primary endpoint was protection against confirmed symptomatic SARS-CoV-2 infection. A total of 10,218 participants were randomly assigned to receive a vaccine or placebo. Virus-neutralizing antibodies were assessed in 419 participants. A dramatic increase (11.3-fold; 128.3–1452.8) of neutralizing titres was measured in the V-01 group at 14 days after the booster. Over two months of surveillance, vaccine efficacy was 47.8% (95%CI: 22.6–64.7) according to the intention-to-treat principle. The most common adverse events were transient, mild-to-moderate pain at the injection site, fever, headache, and fatigue. Serious adverse events occurred almost equally in V-01 (0.12%) and placebo (0.16%) groups. The heterologous boosting with the V-01 vaccine was safe and efficacious, which could elicit robust humoral immunity under the epidemic of the Omicron variant.

**Trial registration:** ClinicalTrials.gov identifier: NCT05096832.

## Introduction

As of 1 March 2022, 435.6 million confirmed cases of COVID-19, including 5.9 million deaths were reported worldwide [1]. To control the virus from raging around the world, vaccines developed with different technologies had high hopes [2]. However, a rapid decrease of neutralizing antibody titres in the first 3-month after the second dose was observed [3], accompanied by a significant decline of protection six months after completion of the two-dose primary regimen [4,5]. A similar decline in neutralizing titres was seen in China-made inactivated vaccines [6,7]. A booster dose is essential, especially heterologous boosting which has demonstrated the ability to restore the immunity in vaccines who have completed primary immunization with viral vector vaccine or mRNA vaccines [8,9]. Although homologous booster effects of inactivated vaccines have been illustrated in clinical trials [6,7], incremental protection of heterologous boosting has not been studied in China-made inactivated vaccine-primed populations. Therefore, we herein reported the efficacy of heterologous boosting using recombinant SARS-CoV-2 fusion protein vaccine (hereinafter referred to as V-01) in those people who received two doses of inactivated vaccines 3–6 months ago.

## Methods

### Vaccine

The V-01 vaccine developed by Livzon Mabpharm Inc. adopts the innovative RBD dimer-IFN-Pan Fc fusion protein molecular design, which can significantly enhance immunogenicity [10]. In this design, RBD is armed with an interferon-α (IFNα) at the N-terminus and dimerized by human IgG1 Fc at the C-terminus (named I-R-F) to target and activate dendritic cells to migrate toward the local draining lymph nodes (LNs), thus enhancing antigen processing and presentation. In addition, a pan HLA-DR-binding epitope (PADRE) is added to I-R-F (named I-P-R-F) to enhance helper T cell response [11]. The fusion protein is expressed in recombinant CHO (Chinese hamster ovary) cells, and the formulation contains 10 μg of fusion protein absorbed in 0.25 mg aluminium hydroxide, 0.66 mg/mL glacial acetic acid, 1.22 mg/mL sodium acetate (equivalent to 20 mM acetate buffer), 44.2 mg/mL trehalose, 0.2 mg/mL polysorbate 80, 4.68 mg/mL sodium chloride, and suspended in 0.5 ml buffer saline. The placebo was identical to the V-01 vaccine except that it did not contain fusion protein.

### Participants and study design

This double-blind, placebo-controlled, randomized phase III clinical trial was conducted in Pakistan and Malaysia and aimed to assess the efficacy, immunogenicity and safety of heterologous boosting with one dose of V-01 vaccine within 3–6 months after the completion of primary vaccination. The protocol was approved by the ethics review committees of each study site, and written informed consent was obtained from each participant before enrolment. An independent data and safety monitoring board reviewed efficacy and safety data. The study was registered with ClinicalTrials.gov (number: NCT05096832).

Adults aged 18 years or older who were healthy or had stable chronic medical conditions in the previous 3-month and completed two doses of inactivated vaccine primary regimen (either BBIBP-CorV produced by China National Biotec Group Company Limited [12] or CoronaVac manufactured by Sinovac Life Sciences [13]) 3–6 months ago were eligible for participation. Exclusion criteria included a medical history of COVID-19, SARS and MERS; a positive SARS-CoV-2 PCR test at baseline; an immunocompromising condition; having received COVID-19 vaccines other than BBIBP-CorV and CornaVac; and pregnant or breastfeeding women. Eligible participants were randomly assigned in a 1:1 ratio to receive one dose of V-01 vaccine or placebo delivered in the deltoid muscle, stratified by age (18–59 years vs ≥ 60 years), gender (male vs female) and brand of inactivated vaccine (BBIBP-CorV vs CoronaVac). Unique allocation numbers were generated by computer and designated to study-agent vials with a block size of eight. After the administration of study agents, all eligible participants were followed actively by automatically sending SMS based on smartphone, telephone call or email once every week.

### Efficacy assessment

The primary endpoint was the protection against confirmed symptomatic SARS-CoV-2 infection with onset at least 14 days after the administration of study agents, and the secondary endpoint was the protection of severe or critical COVID-19. Case definitions were established according to World Health Organization’s (WHO) guidelines [14,15]. Nasopharyngeal or oropharyngeal swabs were required from each suspected case. A SARS-CoV-2 RT–PCR test would be carried out by the Country Reference Laboratory where the study centre was located. For those with confirmed SARS-CoV-2 infection, the weekly follow-up would be performed to assess the severity until recovery, and the PCR-positive samples would be further sequenced at the Wuhan Institute of Virology, Chinese Academy of Sciences. A confirmed symptomatic SARS-CoV-2 infection was defined as positive for SARS-CoV-2 by PCR test at the Country Reference Laboratory in Pakistan or Malaysia and met the following criteria: (a) acute onset of any two or more of the following signs or symptoms, and last for 2 days or more (i.e. ≥48 h): fever, chills, sore throat, nasal obstruction, muscle pain, fatigue, headache, nausea or vomiting, diarrhoea, OR (b) acute onset of any one or more signs or symptoms of the respiratory tract (cough and tachypnoea), onset of anosmia (loss of smell) or ageusia (loss of taste) in the absence of any other identified cause, OR (c) clinical or radiographic evidence of pneumonia if applicable. Endpoint events were judged by an independent adjudication committee that was unaware of group assignment.

A secondary endpoint was the protection of severe or critical COVID-19 defined as a confirmed symptomatic SARS-CoV-2 infection and met one of the following criteria: (a) tachypnoea, respiratory rate ≥ 30 breaths/minute; (b) oxygen saturation [SpO2] ≤ 93% on room air; (c) arterial partial pressure of oxygen/fraction of inspired oxygen [PaO2/FiO2] ≤ 300 mmHg (in the areas with altitude over 1000 metres; (d) progressive worsening of clinical symptoms; chest imaging showing significant lesion progression > 50% within 24–48 h (if applicable); (e) respiratory failure and requiring mechanical ventilation; (f) shock, OR with other organ failures that requires intensive care unit (ICU) care. Endpoint events were judged by an independent adjudication committee that was unaware of group assignment.

### Immunogenicity assessment

A subgroup of participants in this study was randomly selected for the evaluation of immunogenicity. Blood samples were obtained from each selected participant at baseline and on days 14, and 28 after the injection, and the neutralizing activity against ancestral strain was quantified at the Wuhan Institute of Virology, Chinese Academy of Sciences by conducting micro-dose cytopathogenic effect assays, with a limit of detection (LOD) of 10.

#### Live SARS-CoV-2 virus amplification and titration

SARS-CoV-2 virus (BetaCoV/Wuhan/AMMS01/2020 activated, GISAID No. EPI_ISL_5402124) was propagated on Vero E6 cells. The virus was grown until the cytopathic efficiency (CPE) reached > 75% and then harvested. The virus titre was determined by using a CPE assay as follows: 1 × 104 cells/well were seeded in a 96-well culture plate for 18–24 h, after which 10-fold serially diluted virus was added. Six repeats were included for each of the six dilutions. Cells were cultured in a 5% CO2 incubator at 37°C and checked under a microscope for the presence of CPE after 4–5 days. The virus titre was calculated with the Reed and Muench method.

#### Live SARS-CoV-2 neutralization assay

A CPE assay was used to determine the 50% neutralization titre to live SARS-CoV-2. Each serum sample was first incubated at 56°C for 30 min for safety. Vero E6 cells were seeded in a 96-well culture plate for 18–24 h at a density of 1 × 104 cells/well. On the next day, the inactivated serum was serially diluted 3- or 5-fold, starting at 1:5, and six repeats were used for each dilution. In each well of the 96-well plate, 70 µl of serially diluted sera was mixed with 70 µl of 140 TCID50 viruses, then the sera/virus mixture was incubated at 37°C (5% CO2) for 2 h before transferring 100 µl of the sera/virus mixture to 96-well titre plates with confluent Vero E6 cells. After a 4-day incubation, the plate was observed under a microscope and the CPE of each well was recorded. The neutralizing titre was set as the dilution number of the 50% protective condition using the Reed and Muench method.

### Safety assessment

The primary safety endpoints were local or systemic adverse events that occurred within 28 days after the receipt of study agent. The safety observation included close monitoring for immediate adverse events after injection, and reports of the local and systemic reactions were solicited and recorded daily on diary cards within 7 days. Any other symptoms or signs occurring during a 28-day follow-up period were recorded as unsolicited adverse events. A serious adverse event (SAE) was defined as any new health-related problem that resulted in death, was life-threatening, necessitated hospitalization or prolongation of existing hospitalization, or resulted in disability or incapacity. SAEs were recorded throughout the entire study period. Participants were encouraged to contact the investigator immediately in the situation of a serious adverse event.

### Data management and statistical analysis

The sample size was calculated to ensure adequate evaluation of the primary efficacy endpoint. The null hypothesis was that the upper bound of the two-sided 95% confidence interval of hazard ratio (HR) of the V-01 vaccine group compared to the placebo group was larger than 0.7955, which was designed according to the article [16]. This study was designed to be driven by the total number of cases to demonstrate a relative vaccine efficacy (V-01 group vs. placebo group) to prevent confirmed COVID-19. Under the assumption of proportional hazards over time in the two groups, and the expected efficacy against the Delta variant was 26% with a 95% confidence interval of 12–40% in the placebo boosted group and 70% in the V-01 vaccine boosted group, respectively, according to the model [17]. From this, for the sample size calculation in this study, we deduced that the alternative hypothesis regarding the primary endpoint which was PCR-confirmed COVID-19 was that the lower boundary of 95% confidence interval of vaccine efficacy was larger than 20.45%. Two interim analyses would be planned to be conducted when 35% and 70% of the endpoint cases were achieved using Lan-DeMets O'Brien-Fleming approximation spending function for alpha assignment with a total type I error of 2.5%. Based on the group sequential design for time-to-event endpoint, a total of 103 events would provide 90% power. Thus, it was estimated that the expected endpoint cases could be observed if 10,722 participants were enrolled within 3 months with at least 5 months of follow-up period after 14 days post administration of the investigational product, annual incidence rate of 3.2% in the placebo group, dropout rate of 2% per month (loss of evaluable participants) and 15% of participants will be excluded from the PPS. With regard to the sample size required for immunogenicity assessment, assuming a 4-fold increase of geometric mean titres (GMTs) of neutralizing antibody against ancestral strain could be expected in the V-01 group compared to the placebo group, which was equivalent to the difference of 0.60206 after log10 transformation between the two groups. With an assumption of standard deviation (SD) of 0.6 in log10-transformed antibody titres, 200 participants in each group had the statistical power of > 99.9% to detect the difference with a one-sided significance level of 2.5%.

The analysis of incremental efficacy was based on the modified intent-to-treat dataset (mITT), as well as per-protocol dataset (PPS). The mITT dataset included all randomized participants who receive one dose of study agent according to the principle of intent-to-treat (ITT), and who did not experience a confirmed COVID-19 within 14 days after the administration of study agents. PPS included all participants in mITT, who did not experience any major protocol deviations. Incremental efficacy was defined as the percentage reduction in the hazard ratio of the V-01 vaccine group to the placebo group for the confirmed COVID-19. A stratified Cox proportional hazards model adjusting for covariates was applied to estimate the efficacy as well as its confidence interval. The analyses of safety included all randomized participants who received study agent. Those participants with administration errors would be evaluated for safety as per the actual administrative status according to the all participants as treated (ASaT) principle. Safety profiles were summarized according to terms in the Medical Dictionary for Regulatory Activities (MedDRA), version 23.1. The analysis of immunogenicity was performed also according to the principle of ASaT. All participants who participated in the immunogenicity subgroup with valid immunogenicity data were included. GMTs of neutralizing antibodies and their 95% confidence intervals were calculated. Antibody titres were logarithmically converted to allow assessment of GMTs. The SAS programme (SAS Institute Inc., Cary, NC, USA, version 9.4) was employed for statistical analysis. A *p*-value < 0.05 (two-tailed) was considered statistically significant.

## Results

### Summary of participants

**Figure 1.**
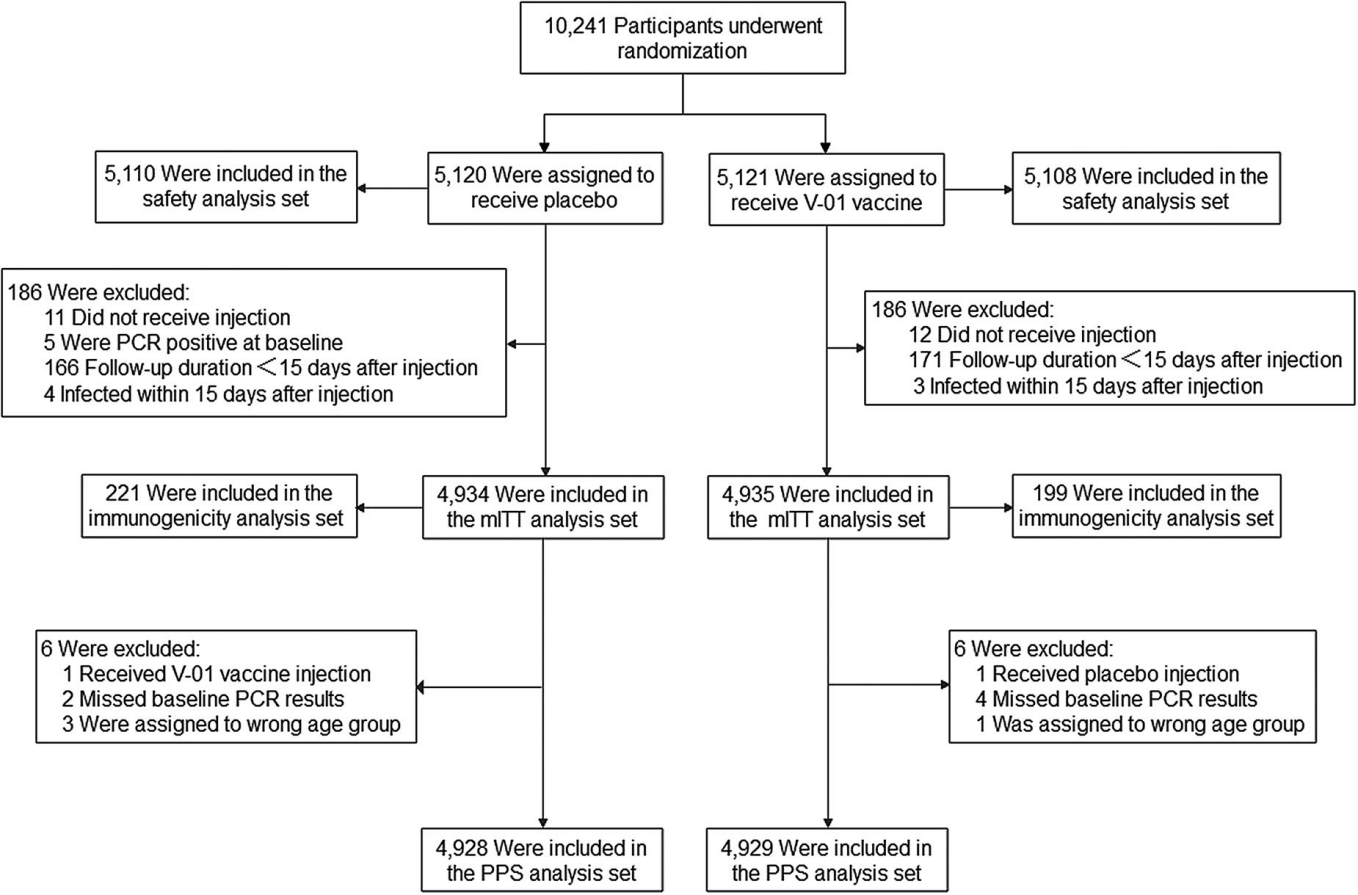
Summary of participants. The primary efficacy analysis was performed based on the modified intent-to-treat (mITT) set. A total of 10,241 eligible participants were randomly assigned to receive V-01 vaccine or placebo. After a 2-month follow-up, 4,935 participants in the V-01 vaccine group and 4,934 in the placebo group were included in the MITT. Of these, 199 and 221 participants in V-01 vaccine and placebo group, respectively, formed the immunogenicity analysis set, blood samples were obtained at baseline and at days 14 and 28 to assess the anti-SARS-CoV-2 neutralizing antibody titres.

Between 3 November 2021 and 27 January 2022, 10,863 participants were screened for eligibility. Of these, 5,121 and 5,120 eligible participants who all were Asian ethnicity were recruited from Pakistan (8,008 participants) and Malaysia (2,233 participants) and randomized to receive the V-01 vaccine and placebo, respectively (Figure 1). A median follow-up duration of 60 days (range, 17–86 days) after booster was calculated in participants included in the mITT dataset. The baseline characteristics of the participants in V-01 vaccine and placebo groups were comparable (Table 1). Although participants primed with BBIBP-CorV and CoronaVac were assigned equally to placebo and V-01 groups, more participants primed by BBIBP-CorV vaccine received booster ≥ 4.5 months (37.6% with BBIBP-CorV vs. 27.2% with CoronaVac, *p* < 0.001). Furthermore, a longer average interval between the first and second doses of the primary course was found in participants primed with the BBIBP-CorV vaccine (47.1 days with a standard deviation of 30.7 days) compared to those with CoronaVac vaccine (37.3 days with a standard deviation of 15.8 days) (*p* < 0.001). The proportions of the elderly in the BBIBP-CorV and CoronaVac groups were equal (5.2% vs 5.2%, respectively).

**Table 1.** Characteristics of participants at baseline according to ITT principle.

Characteristics	Placebo group (*N* = 4934)	V-01 group (*N* = 4935)	Total (*N* = 9869)
Gender (n, %)			
Male	3535 (71.7)	3538 (71.7)	7073 (71.7)
Female	1399 (28.3)	1397 (28.3)	2796 (28.3)
Mean age (range) — yr	36.7 (18.0, 82.0)	36.8 (18.0, 82.0)	36.8 (18.0, 82.0)
Age group (n, %)			
18–59 yr	4675 (94.8)	4677 (94.8)	9352 (94.8)
≥60 yr	259 (5.2)	258 (5.2)	517 (5.2)
Nationality (n, %)			
Pakistan	3984 (80.7)	4003 (81.1)	7987 (80.9)
Malaysia	950 (19.3)	932 (18.9)	1882 (19.1)
Body-mass index ≥ 30.0 (n, %)	852 (17.3)	844 (17.1)	1696 (17.2)
Brand of vaccines used in primary vaccination (n, %)			
BBIBP-CorV	1315 (26.7)	1316 (26.7)	2631 (26.7)
CoronaVac	3619 (73.3)	3619 (73.3)	7238 (73.3)
Interval between primary and booster			
Mean days (SD)	122.1 (24.7)	122.6 (24.9)	122.3 (24.8)
Vulnerable participants (n, %)			
Yes	582 (11.8)	557 (11.3)	1139 (11.5)
No	4352 (88.2)	4378 (88.7)	8730 (88.5)

### Efficacy

**Figure 2.**
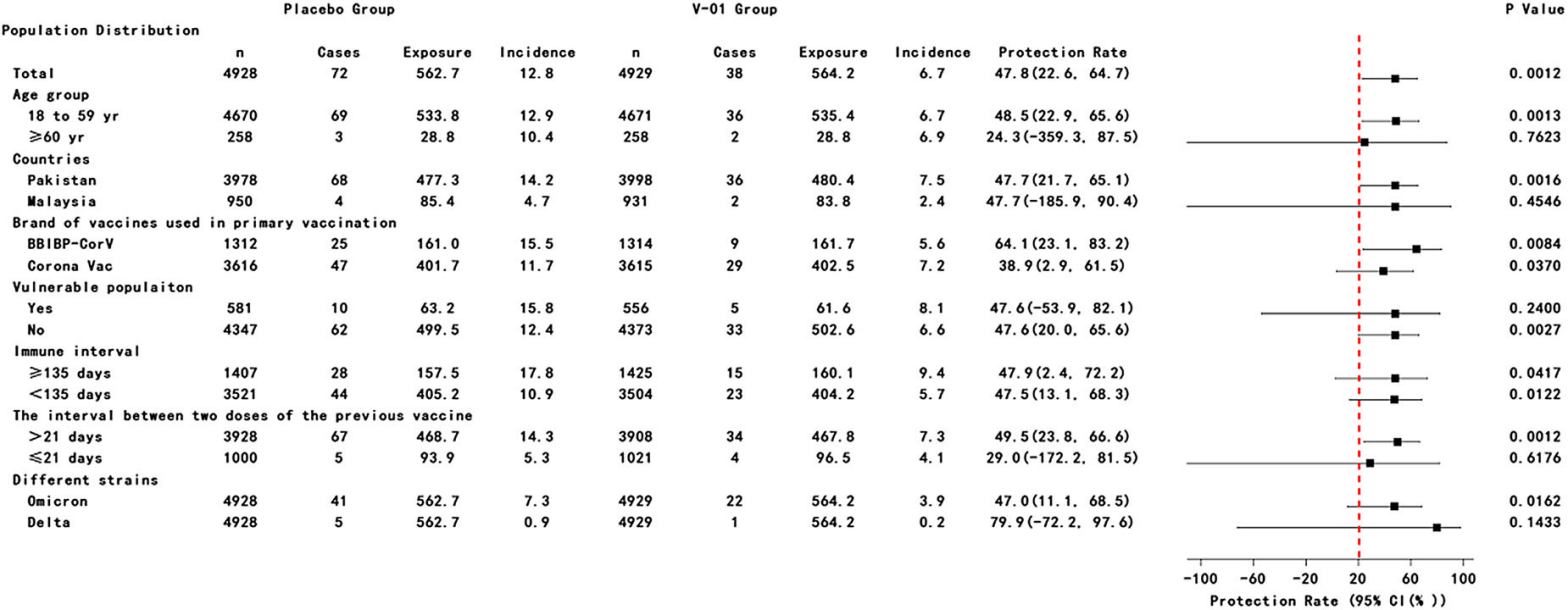
Efficacy of heterologous boost with V-01 vaccine preventing COVID-19 in subgroup according to mITT analysis. Vaccine efficacy was defined as the percentage reduction in the hazard ratio of V-01 vaccine group to the placebo group for the confirmed COVID-19. A stratified Cox proportional hazards model adjusting for covariates was applied to estimate the efficacy as well as its confidence interval.

Between day 1 after injection and 27 January 2022, a total of 275 suspected SARS-CoV-2 symptomatic infections were reported through active disease surveillance. After the assessment, 117 PCR-confirmed COVID-19 cases were identified. Of these, 7 COVID-19 cases presented within 14 days after injection (4 cases in placebo group vs 3 cases in V-01 vaccine group). For the primary endpoint analysis, 72 and 38 PCR-confirmed COVID-19 cases were determined from the placebo and V-01 vaccine groups, and resulted in an incremental efficacy of 47.8% (95%CI: 22.6–64.7) and 47.8% (95%CI: 22.6–64.8) in terms of mITT and PPS analysis, respectively (Figure 2 and Figure S1). In accordance with the ITT principle, the further stratified analysis indicated that higher efficacy was found in those participants aged 18–59 years (48.5%), primed with BBIBP-CorV vaccine (64.0%), compared to those who ≥ 60-year (24.1%), primed with CoronaVac vaccine (38.9%), while the protections had no difference among other subgroups (Figure 2). A similar profile of protection against symptomatic SARS-CoV-2 infection was also obtained in terms of per-protocol analysis (Figure S1). Deep sequencing was performed with all 110 PCR-positive specimens, and the results for 69 specimens were valid. Of these, 63 (91.3%) specimens were identified as Omicron variant infection (41 vs 22 Omicron infections in placebo and V-01 groups respectively), resulting in an efficacy of 47.0% (95%CI: 11.1–68.4). Comparatively, the efficacy (79.9%; 95%CI: −72.0–97.7) against the Delta variant was higher (Figure 2). The protection against suspected COVID-19 with a negative PCR test was −20.7% (95%CI: −65.1% to 11.8%, *p* > 0.05), while the secondary endpoint of preventing severe disease was not able to be assessed due to the limited number of cases.

### Immunogenicity

**Figure 3.**
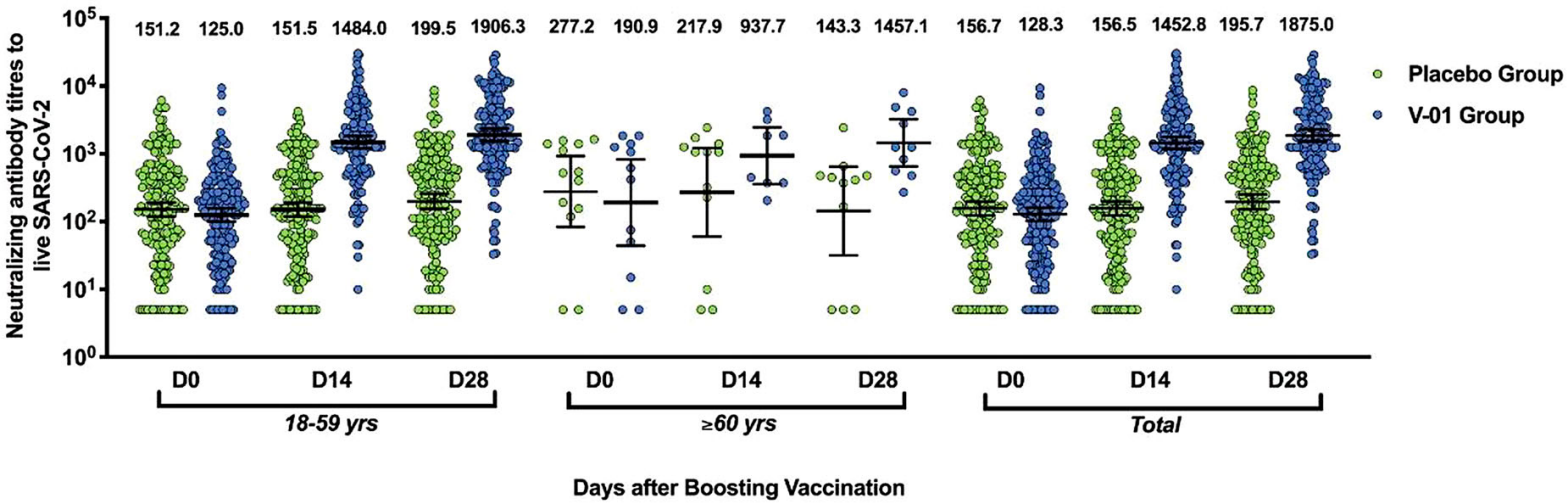
Virus-neutralizing titres pre- and post-booster. Serum was obtained before booster (day 0), 14, and 28 days after the booster vaccination. The neutralizing activity against ancestral strain was quantified by micro-dose cytopathogenic effect assays, with a limit of detection (LOD) of 10. The number above the scatter bars is the GMT for the group.

At the baseline, significant differences in neutralizing antibodies positivity and antibodies titres were not detected between the V-01 vaccine and placebo groups, in terms of the age, gender, study countries (Pakistan and Malaysia), and brand of prime vaccines (Table S2). Overall, at the baseline, the neutralizing antibodies positive rate was 93.9% and 91.4% (*p* > 0.05), with a GMT of 128.3 and 156.7 (*p* > 0.05) in V-01 vaccine and placebo groups, respectively. A dramatic increase (11.3-fold; 128.3–1452.8) of neutralizing titres was measured in the V-01 vaccine group, rather than the placebo group at 14 days after the booster. Two more weeks later, a GMT of 1875.0 was approached in the V-01 vaccine group, compared to a GMT of 195.7 in the placebo group (Figure 3).

### Safety

**Figure 4.**
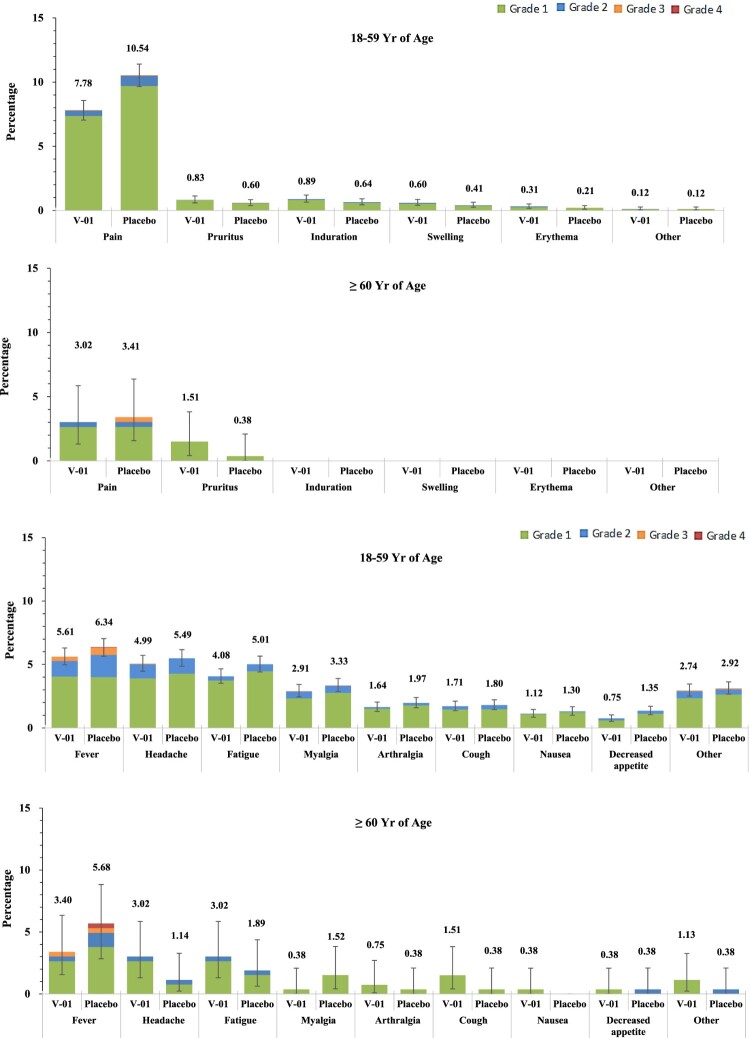
Solicited Local and Systemic Adverse Events. Solicited local and systemic reactions were collected by diary cards within 7 days after booster from participants in the safety analysis set (5,108 and 5,110 participants in V-01 and placebo group, respectively). Solicited local reactions are shown in Panel A, and solicited systemic reactions are shown in Panel B. Each vertical bar represents the percentage of participants who reported the specified reaction with a 95% confidence interval.

The overall occurrence rate of adverse events was 19.7% and 21.5% in V-01 and placebo groups respectively, and the majority of adverse events were defined as grade 1 and grade 2 (98.8 in the V-01 group vs 98.4% in the placebo group). The injection-site pain was the most common local adverse event (7.5% in V-01 vaccine and 10.2% in placebo groups, respectively), followed by itching and induration (occurrences were all lower than 1%), while fever (5.5% in V-01 group vs 6.3% in placebo group), headache (4.9% in V-01 group vs 5.3% in placebo group) and fatigue (4.0% in V-01 group vs 4.9% in placebo group) were reported more frequently as systemic adverse events (Figure 4). A total of 14 serious adverse events were reported; of these, 6 (0.12%) and 8 (0.16%) events occurred in V-01 and placebo groups, respectively (*p* > 0.05), and only 1 event (occurred at 8 weeks after injection, and diagnosed as severe autoimmune thrombocytopenia) was considered possibly related to injection in the placebo group. There was no report on adverse events of special interest during the study period.

Vaccination-associated safety profiles, including solicited and unsolicited adverse reactions in V-01 vaccine and placebo groups, were balanced (Table S3). Within 7 days after injection, solicited adverse reactions presented almost equally in V-01 vaccine and placebo groups (17.0% vs. 18.8%, *p* > 0.05). Compared to the elderly (≥60 years), adults aged 18 and 59 tended to have more solicited adverse reactions in both groups, including local and systemic reactions (Table S4 and S5), while this age-related difference was not observed in the occurrence of an unsolicited adverse event. The local adverse reactions were mainly grades 1 (8.8%) and grade 2 (0.7%) in severity. Again, the most commonly reported solicited systemic adverse reactions were fever (5.5% in V-01 vaccine and 6.3% in placebo groups), followed by headache, fatigue, and myalgia (4.9%, 4.0%, 2.8% and 5.3%, 4.9%, 3.2% in V-01 vaccine and placebo groups respectively). The majority of systemic adverse reactions reported were mild (12.0% in grade 1 and 3.4% in grade 2), and were reported more frequently by adult participants than by elderly (Figure 4, Tables S4 and S5).

## Discussion

Concerns over waning immunity and SARS-CoV-2 variants co-motivated the need for an extra dose [18,19]. Our study demonstrated that a heterologous boosting using recombinant SARS-CoV-2 fusion protein vaccine among those people primed with two doses of inactivated vaccines was safe and conferred a promising incremental efficacy of 47.8%, which met our pre-specified success criteria. Moreover, a better efficacy (79.9%) against the Delta variant was observed, though the statistical significance was not reached due to the limited prevalence in the study population. Although an enhanced humoral immunity of homologous or heterologous booster based on two-dose of inactivated vaccines has been validated in animal studies, as well as human trials [7,20,21], the efficacy conferred by a heterologous boosting was first demonstrated. Hyporesponsiveness in elderly has been reported in clinical studies of the COVID-19 vaccine [22–24]. As anticipated, a lower efficacy in participants ≥ 60 years was observed in our study. Due to immunosenescence, age-related changes influence the host immune response, resulting in a weakened ability to fight respiratory infections and hyporesponsiveness to vaccinations [25,26]. Surprisingly, there was a difference in protection between participants primed with BBIBP-CorV and CoronaVac vaccines. The most likely explanation might be the prolonged interval between first and second injections, as well as 2nd and the booster dose. Indeed, a better neutralizing titres elicited by a longer interval of primary and homologous boost with inactivated vaccines, as well as that of heterologous boost with protein subunit vaccine had been reported [7,27,28]. Moreover, such an association between prolonged injection interval and protection was also observed with ChAdOx1 vaccine [29].

It is well known that heterologous boost can be more immunogenic than homologous boost and can increase the intensity, sustainability and breadth of immune responses [30], and thus can potentially provide better protection against variants of concern (VOCs) [31,32]. As expected, heterologous V-01 vaccine boost in inactivated vaccine-primed participants elicited a robust virus-neutralizing antibody response, with a GMT of 1452.8 at the 14 days after boosting, which increased 2.2- and 8.7-fold, respectively, compared to that elicited by homologous boosting of V-01 or CoronaVac vaccines [7,33].

Heterologous boost has risen safety and reactogenicity concern, since several studies reported preliminary analysis on reactogenicity of various heterologous prime-boost regimens using licenced COVID-19 vaccines and indicated a clearly increased reactogenicity after heterologous boost with BNT162b2 in ChAdOx1-primed participants [34,35], whereas, in our study, the safety profile was superior to that of primary and boosting administrated with inactivated vaccines or V-01 vaccine alone [7,21,33]. Nevertheless, no matter what kind of heterologous regimens, the overall reactogenicity was tolerable and manageable, when considering the essential incremental protection against VOCs.

The protection estimated in our study might be influenced. First, hybrid immunity was measured in our study. At the baseline (within 3–6 months after primary regimen), the neutralizing titres was around 150 in V-01 vaccine and placebo groups, which was remarkably higher than the neutralizing titres at the same time point after primary regimen using BBIBP-CorV or CoronaVac vaccines studied in China [6,7]. The reason behind was the possibility of mixture with natural infection could be ruled out in these studies carried out in China, which under the “Zero COVID-19” circumstance. Moreover, within 28 days after receiving the placebo injection in our study, the neutralizing titres also increased ∼25% (from 156.7 to 195.7). Clearly, natural infection occurred and persisted all the time in the study population. A recent study indicated that when natural immunity was combined with vaccine-generated immunity, 25- to 100-fold higher antibody responses could approach, driven by memory B cells and CD4+ T cells and broader cross-protection from variants, regardless of whether it occurs before or after vaccination [36,37]. Thus, hybrid immunity narrowed the gap in incidence between the two groups and led to an underestimation of protection. Second, compared to previous variants, the SARS-CoV-2 Omicron variant harbours 34 mutations in the spike and thus can more efficiently evade immunity elicited by vaccination [38–40], which might weaken the protection of current available COVID-19 vaccines, while our study overlapped with the epidemic of Omicron variants.

Our study has several limitations. First, due to the sharp occurrence of symptomatic infection caused by the Omicron variant, the present number of primary endpoint events was hit quickly in three months, the duration of efficacy follow-up was relatively short, whereas, the trial is ongoing according to a 20-month follow-up plan that will allow assessment of protective duration. Second, only 5.2% of the elderly were recruited which resulted in a wide 95% confidence interval around the point estimate, and pregnant women were excluded from this trial; extrapolation of findings to subpopulations other than 18–59 years requires caution or further evidence. Third, because of the mild clinical manifestations after Omicron infection [41], the characteristics to prevent severe disease were not able to be defined. Finally, relative efficacy of V-01 heterologous boost versus placebo boost in participants primed with a 2-dose inactivated vaccine was measured in this study, while absolute efficacy would be more informative to convey the real magnitude of protection. Whereas, it become impossible given the coverage and regulatory guidelines, and thus, the absolute efficacy could only be speculated based on the published efficacies of the two-dose primary regimen. Currently, only one phase III trial reported efficacy of 65.3% over 2.5 months surveillance period before the waves of Delta and Omicron variants [42]. In addition, the placebo boost, rather than the homologous boost, was included in the current study for comparison, thereby leaving room for reconsideration of whether the heterologous boost strategy could provide better protection under the raging of the Omicron variant. There are a couple of reasons behind. First, at the time of the designing trial (October 2021), for inactivated vaccines, the need for, and timing of booster dose, was being assessed by regulatory authorities, including WHO [43]. To be solid evidence, measurement of absolute incremental efficacy provided by the V-01 boost was extremely desirable. Secondly, in practice, it was not feasible to obtain enough amount of inactivated vaccines, and distribute them blindly to the right participants. Whereas, it is indeed a limitation at this stage that the need for a second dose is being assessed. Nevertheless, a small trial aimed to compare the immune response elicited by heterologous and homologous boosts has been completed recently in China. The preliminary results indicated that the pseudovirus neutralizing antibodies titres against the Omicron variant was 3.7 folds higher in the heterologous V-01 boost group compared to that in the homologous inactivated boost group, and the cellular immune responses, including T cell immune responses, are being analyzed (data will be published in another article).

In conclusion, the V-01 vaccine that adopted the innovative prototype-sequenced RBD dimer-INF-Pan Fc fusion protein molecular design was safe, efficacious, and could elicit robust humoral immunity when boosted in inactivated vaccine-primed participants during the pandemic of the Omicron variant. Findings from this heterologous boost trial should be considered to further optimize the immunization schedule and strategy for the control of COVID-19.

## Authors contributions

SFM, WKC, WXG, XY and XYW conceived the trials, FJ, WD, ZHX, JMY, SFM, WKC, and WXG contributed to the protocol of the study. XYW is the chief investigator. SFM and WKC are principal investigators in Pakistan and Malaysia. SFM, WKC, MA, MAS, WA, VKS, MAS, SL and AHT contributed to the implementation of the study or data collection. LYL and WXG were responsible for neutralizing antibody testing and analysis. YYQ, WD, ZHX, JMY, YZ and XYW accessed and verified the data. FJ, YYQ, WD, YZ, XY, and XYW contributed to the preparation of the report. All authors critically reviewed and approved the final version. All authors had full access to all the data in the study and had final responsibility for the decision to submit for publication.

## Supplementary Material

Supplemental Material
