# Supplementary material for: Efficacy of heterologous boosting against SARS-CoV-2 using a recombinant interferon-armed fusion protein vaccine (V-01): a randomized, double-blind and placebo-controlled phase III trial
Source: Emerg Microbes Infect. 2022 Aug 1;11(1):1910–9. doi: 10.1080/22221751.2022.2088406 (PMC9347473; doi:10.1080/22221751.2022.2088406)
Supplement: Supplemental Material [file TEMI_A_2088406_SM8881.docx]

**Table S1.** Characteristics of participants at baseline according to PP principle

| Characteristics | Placebo Group  (N= 4928) | V-01 Group  (N= 4929) | Total  (N= 9857) |
| --- | --- | --- | --- |
| Sex — no. of participants (%) |  |  |  |
| Male | 3531 (71.7) | 3532 (71.7) | 7063 (71.7) |
| Female | 1397 (28.3) | 1397 (28.3) | 2794 (28.3) |
| Mean age (range) — yr | 36.7 (18.0, 82.0) | 36.8 (18.0, 82.0) | 36.8 (18.0, 82.0) |
| Age group (%) |  |  |  |
| 18 to 59 yr | 4670 (94.8) | 4671 (94.8) | 9341 (94.8) |
| ≥60 yr | 258 (5.2) | 258 (5.2) | 516 (5.2) |
| Body-mass index≥30.0 (n, %) | 850 (17.2) | 843 (17.1) | 1693 (17.2) |
| Brand of vaccines used in primary vaccination (n, %) | | |  |
| BBIBP-CorV | 1312 (26.6) | 1314 (26.7) | 2626 (26.6) |
| CoronaVac | 3616 (73.4) | 3615 (73.3) | 7231 (73.4) |
| Vaccination interval between primary and booster | | |  |
| Mean days (SD) | 122.1 (24.7) | 122.6 (24.9) | 122.3 (24.8) |
| Vulnerable population (n, %) |  |  |  |
| Yes | 581 (11.8) | 556 (11.3) | 1137 (11.5) |
| No | 4347 (88.2) | 4373 (88.7) | 8720 (88.5) |

**Table S2**. Characteristics of anti-SARS-CoV-2 neutralizing antibody at baseline

| Characteristics | Placebo group | | | | | V-01 vaccine group | | | |  |
| --- | --- | --- | --- | --- | --- | --- | --- | --- | --- | --- |
|  | No. of participants | Positive rate (95%CI) | | | GMT (95% CI) | No. of participants | Positive rate (95%CI) | | GMT (95% CI) |  |
| Total | 221 | 91.4% (86.9-94.7) | | | 156.7 (124.0-198.1) | 198 | 93.9% (89.6-96.8) | | 128.3 (102.5-160.5) |  |
| Age group | | |  |  | | | |  | | |
| 18 to 59 yr | 208 | 91.8% (87.2-95.2) | | | 151.2 (119.0-192.2) | 186 | 94.6% (90.3-97.4) | | 125.0 (99.9-156.5) |  |
| ≥60 yr | 13 | 84.6% (54.6-98.1) | | | 277.2 (83.0-925.9) | 12 | 83.3% (51.6-97.9) | | 190.9 (44.1-825.9) |  |
| Gender |  |  | | |  |  |  | |  |  |
| Male | 162 | 88.9% (83.0-93.3) | | | 157.2 (117.9-209.5) | 144 | 95.1% (90.2-98.0) | | 148.4 (116.2-189.5) |  |
| Female | 59 | 98.3% (90.9-100.0) | | | 155.4 (104.4-231.3) | 54 | 90.7% (79.7-96.9) | | 87.0 (52.8-143.6) |  |
| Countries | | |  |  | | | |  | | |
| Pakistan | 189 | 93.1% (88.5-96.3) | | | 202.7 (158.8-258.9) | 166 | 96.4% (92.3-98.7) | | 173.7 (139.3-216.7) |  |
| Malaysia | 32 | 81.2% (63.6-92.8) | | | 34.2 (21.2-55.3) | 32 | 81.2% (63.6-92.8) | | 26.6 (15.6-45.3) |  |
| Brand of vaccines used in primary vaccination | | | | | | | |  | | |
| BBIBP-CorV | 80 | 91.2% (82.8-96.4) | | | 182.1 (123.4-268.7) | 69 | 92.8% (83.9-97.6) | | 155.5 (107.0-226.0) |  |
| CoronaVac | 141 | 91.5% (85.6-95.5) | | | 143.9 (107.0-193.5) | 129 | 94.6% (89.1-97.8) | | 115.8 (87.3-153.5) |  |

**Table S3.** Adverse Reactions (ARs)and Adverse Reactions of Grade 3 or Higher

| ARs | All Adverse Reactions | | | Adverse Reactions of Grade 3 or Higher | | |
| --- | --- | --- | --- | --- | --- | --- |
|  | Placebo Group (N=5110) | V-01 Group (N=5108) | P value | Placebo Group (N=5110) | V-01 Group (N=5108) | P value |
|  | no. of participants with event (%) | |  | no. of participants with event (%) | |  |
| ns | 5093 | 5074 |  | 5093 | 5074 |  |
| Solicited Adverse Reactions ＜7 days after injection | 956 (18.77) | 861 (16.97) | 0.0185 | 39 (0.77) | 26 (0.51) | 0.1348 |
| Systemic ARs | 728 (14.29) | 682 (13.44) | 0.2173 | 36 (0.71) | 24 (0.47) | 0.1538 |
| Fever | 321 (6.30) | 279 (5.50) | 0.0922 | 31 (0.61) | 17 (0.34) | 0.0589 |
| Headache | 268 (5.26) | 248 (4.89) | 0.3911 | 0 | 3 (0.06) | 0.1243 |
| Fatigue | 247 (4.85) | 204 (4.02) | 0.0431 | 0 | 0 | - |
| Myalgia | 165 (3.24) | 141 (2.78) | 0.182 | 1 (0.02) | 2 (0.04) | 0.6243 |
| Arthralgia | 96 (1.88) | 81 (1.60) | 0.2885 | 0 | 0 | - |
| Cough | 88 (1.73) | 86 (1.69) | 0.9391 | 0 | 0 | - |
| Nausea | 63 (1.24) | 55 (1.08) | 0.5171 | 0 | 0 | - |
| Decreased appetite | 66 (1.30) | 37 (0.73) | 0.0053 | 0 | 0 | - |
| Diarrhoea | 49 (0.96) | 38 (0.75) | 0.2816 | 2 (0.04) | 1 (0.02) | 1 |
| Pruritus | 32 (0.63) | 31 (0.61) | 1 | 0 | 0 | - |
| Dyspnoea | 27 (0.53) | 26 (0.51) | 1 | 2 (0.04) | 1 (0.02) | 1 |
| Dysphagia | 24 (0.47) | 22 (0.43) | 0.8827 | 0 | 0 | - |
| Constipation | 21 (0.41) | 23 (0.45) | 0.7651 | 0 | 0 | - |
| Vomiting | 19 (0.37) | 16 (0.32) | 0.7355 | 0 | 0 | - |
| Mucocutaneous disorder | 15 (0.29) | 13 (0.26) | 0.8504 | 0 | 1 (0.02) | 0.4991 |
| Hypersensitivity | 7 (0.14) | 7 (0.14) | 1 | 0 | 0 | - |
| Local ARs | 537 (10.54) | 413 (8.14) | <0.0001 | 4 (0.08) | 2 (0.04) | 0.6874 |
| Pain | 518 (10.17) | 382 (7.53) | <0.0001 | 3 (0.06) | 2 (0.04) | 1 |
| Pruritus | 30 (0.59) | 44 (0.87) | 0.1035 | 1 (0.02) | 0 | 1 |
| Induration | 31 (0.61) | 43 (0.85) | 0.1632 | 0 | 0 | - |
| Swelling | 20 (0.39) | 29 (0.57) | 0.2008 | 0 | 0 | - |
| Erythema | 10 (0.20) | 15 (0.30) | 0.3255 | 0 | 0 | - |
| Rash | 6 (0.12) | 5 (0.10) | 1 | 0 | 0 | - |
| Cellulitis | 0(0.00) | 2(0.04) | 0.249 | 0 | 0 | - |
| Unsolicited Adverse Reactions ＜28 days after injection | 58 (1.14) | 45 (0.88) | 0.2345 | 0 | 2 (0.04) | 0.2499 |
| Respiratory, thoracic and mediastinal disorders | 34 (0.67) | 28 (0.55) | 0.5245 | 0 | 0 | - |
| Oropharyngeal pain | 19 (0.37) | 14 (0.27) | 0.4861 | 0 | 0 | - |
| Cough | 21 (0.41) | 11 (0.22) | 0.1096 | 0 | 0 | - |
| Nasal obstruction | 8 (0.16) | 9 (0.18) | 0.8143 | 0 | 0 | - |
| Tachypnoea | 4 (0.08) | 4 (0.08) | 1 | 0 | 0 | - |
| Rhinorrhoea | 3 (0.06) | 1 (0.02) | 0.6249 | 0 | 0 | - |
| Dyspnoea | 0 | 2 (0.04) | 0.2499 | 0 | 0 | - |
| Productive cough | 0 | 1 (0.02) | 0.4999 | 0 | 0 | - |
| Sneezing | 1 (0.02) | 0 | 1 | 0 | 0 | - |
| General disorders and administration site conditions | 31 (0.61) | 21 (0.41) | 0.2104 | 0 | 0 | - |
| Fever | 18 (0.35) | 14 (0.27) | 0.596 | 0 | 0 | - |
| Fatigue | 12 (0.23) | 7 (0.14) | 0.3588 | 0 | 0 | - |
| Chills | 2 (0.04) | 4 (0.08) | 0.4529 | 0 | 0 | - |
| Pain | 3 (0.06) | 1 (0.02) | 0.6249 | 0 | 0 | - |
| Chest pain | 2 (0.04) | 0 | 0.5 | 0 | 0 | - |
| Vaccination site pain | 0 | 2 (0.04) | 0.2499 | 0 | 0 | - |
| Asthenia | 1 (0.02) | 0 | 1 | 0 | 0 | - |
| Swelling | 1 (0.02) | 0 | 1 | 0 | 0 | - |
| Injection site pain | 0 | 1 (0.02) | 0.4999 | 0 | 0 | - |
| Musculoskeletal and connective tissue disorders | 16 (0.31) | 14 (0.27) | 0.8553 | 0 | 1 (0.02) | 0.4999 |
| Myalgia | 15 (0.29) | 12 (0.23) | 0.7007 | 0 | 1 (0.02) | 0.4999 |
| Back pain | 0 | 1 (0.02) | 0.4999 | 0 | 0 | - |
| Myokymia | 0 | 1 (0.02) | 0.4999 | 0 | 0 | - |
| Musculoskeletal pain | 1 (0.02) | 0 | 1 | 0 | 0 | - |
| Neck pain | 1 (0.02) | 0 | 1 | 0 | 0 | - |
| Nervous system disorders | 9 (0.18) | 5 (0.10) | 0.4236 | 0 | 0 | - |
| Headache | 7 (0.14) | 5 (0.10) | 0.7743 | 0 | 0 | - |
| Dysgeusia | 1 (0.02) | 1 (0.02) | 1 | 0 | 0 | - |
| Parosmia | 2 (0.04) | 0 | 0.5 | 0 | 0 | - |
| Dizziness | 1 (0.02) | 0 | 1 | 0 | 0 | - |
| Ageusia | 1 (0.02) | 0 | 1 | 0 | 0 | - |
| Gastrointestinal disorders | 5 (0.10) | 7 (0.14) | 0.5808 | 0 | 0 | - |
| Diarrhoea | 3 (0.06) | 1 (0.02) | 0.6249 | 0 | 0 | - |
| Nausea | 2 (0.04) | 1 (0.02) | 1 | 0 | 0 | - |
| Abdominal pain | 1 (0.02) | 1 (0.02) | 1 | 0 | 0 | - |
| Vomiting | 2 (0.04) | 0 | 0.5 | 0 | 0 | - |
| Abdominal distension | 0 | 1 (0.02) | 0.4999 | 0 | 0 | - |
| Hypoaesthesia oral | 0 | 1 (0.02) | 0.4999 | 0 | 0 | - |
| Abdominal pain upper | 0 | 1 (0.02) | 0.4999 | 0 | 0 | - |
| Tongue ulceration | 0 | 1 (0.02) | 0.4999 | 0 | 0 | - |
| Infections and infestations | 6 (0.12) | 5 (0.10) | 1 | 0 | 1 (0.02) | 0.4999 |
| Influenza | 5 (0.10) | 4 (0.08) | 1 | 0 | 0 | - |
| Rhinitis | 1 (0.02) | 0 | 1 | 0 | 0 | - |
| Gastroenteritis | 0 | 1 (0.02) | 0.4999 | 0 | 1 (0.02) | 0.4999 |
| Skin and subcutaneous tissue disorders | 3 (0.06) | 0 | 0.2499 | 0 | 0 | - |
| Dermatitis allergic | 1 (0.02) | 0 | 1 | 0 | 0 | - |
| Rash | 1 (0.02) | 0 | 1 | 0 | 0 | - |
| Pruritus | 1 (0.02) | 0 | 1 | 0 | 0 | - |
| Vascular disorders | 1 (0.02) | 1 (0.02) | 1 | 0 | 0 | - |
| Hypotension | 1 (0.02) | 0 | 1 | 0 | 0 | - |
| Haematoma | 0 | 1 (0.02) | 0.4999 | 0 | 0 | - |
| Injury, poisoning and procedural complications | 0 | 1 (0.02) | 0.4999 | 0 | 0 | - |
| Contusion | 0 | 1 (0.02) | 0.4999 | 0 | 0 | - |
| Reproductive system and breast disorders | 1 (0.02) | 0 | 1 | 0 | 0 | - |
| Vaginal discharge | 1 (0.02) | 0 | 1 | 0 | 0 | - |

1. ns=Number of exposed participants who submitted any data for the event; for solicited adverse reactions ＜7 days after injection, percentages are based on the number of exposed participants who submitted any data for the event in the solicited safety set. N=Number of exposed participants in the Overall Safety Set; for other percentages are based on the number of exposed participants who submitted any data for the event in the Overall Safety Set.
2. Adverse reactions(AR) are defined as any harmful or unintended reactions possibly attributed to the use of investigational vaccine during the clinical trial. If the causal relationship between investigational vaccine and AE implies at least a reasonable possibility, i.e., relevance cannot be excluded.
3. Solicited adverse reactions are ARs that occur within 7 days after vaccination (i.e., the recruitment period) and are pre-listed in the protocol, case report forms and diary cards, which are usually the pre-specified adverse events after vaccination. The solicited ARs can be classified as inoculation site (local) adverse reactions and non-inoculation site (systemic) adverse reactions based on the site.
4. Unsolicited adverse reactions are ARs other than those designated as solicited ARs, and also include eponymous solicited ARs that occur after the solicitation period.

**Table S4.** Solicited Adverse Reactions Within 7 Days After Vaccination by Grade, Solicited Safety Set

| Adverse reaction n (%) | Overall Safety Set | | | ≥18-<60 years | | | ≥60 years | | |
| --- | --- | --- | --- | --- | --- | --- | --- | --- | --- |
|  | Placebo Group | V-01 Group | Total | Placebo Group | V-01 Group | Total | Placebo Group | V-01 Group | Total |
|  | (N=5093) | (N=5074) | (N=10167) | (N=4829) | (N=4809) | (N=9638) | (N=264) | (N=265) | (N=529) |
| Any solicited AR | 956 (18.77) | 861 (16.97) | 1817 (17.87) | 927 (19.20) | 838 (17.43) | 1765 (18.31) | 29 (10.98) | 23 (8.68) | 52 (9.83) |
| Grade 1 | 869 (17.06) | 780 (15.37) | 1649 (16.22) | 845 (17.50) | 761 (15.82) | 1606 (16.66) | 24 (9.09) | 19 (7.17) | 43 (8.13) |
| Grade 2 | 208 (4.08) | 173 (3.41) | 381 (3.75) | 204 (4.22) | 169 (3.51) | 373 (3.87) | 4 (1.52) | 4 (1.51) | 8 (1.51) |
| Grade 3 | 36 (0.71) | 26 (0.51) | 62 (0.61) | 34 (0.70) | 25 (0.52) | 59 (0.61) | 2 (0.76) | 1 (0.38) | 3 (0.57) |
| Grade 4 | 3 (0.06) | 0 | 3 (0.03) | 2 (0.04) | 0 | 2 (0.02) | 1 (0.38) | 0 | 1 (0.19) |
| Any Local AR | 537 (10.54) | 413 (8.14) | 950 (9.34) | 528 (10.93) | 404 (8.40) | 932 (9.67) | 9 (3.41) | 9 (3.4) | 18 (3.4) |
| Grade 1 | 500 (9.82) | 396 (7.80) | 896 (8.81) | 493 (10.21) | 388 (8.07) | 881 (9.14) | 7 (2.65) | 8 (3.02) | 15 (2.84) |
| Grade 2 | 44 (0.86) | 28 (0.55) | 72 (0.71) | 43 (0.89) | 27 (0.56) | 70 (0.73) | 1 (0.38) | 1 (0.38) | 2 (0.38) |
| Grade 3 | 4 (0.08) | 2 (0.04) | 6 (0.06) | 3 (0.06) | 2 (0.04) | 5 (0.05) | 1 (0.38) | 0 | 1 (0.19) |
| Grade 4 | 0 | 0 | 0 | 0 | 0 | 0 | 0 | 0 | 0 |
| Local AR |  |  |  |  |  |  |  |  |  |
| Pain | 518 (10.17) | 382 (7.53) | 900 (8.85) | 509 (10.54) | 374 (7.78) | 883 (9.16) | 9 (3.41) | 8 (3.02) | 17 (3.21) |
| Grade 1 | 475 (9.33) | 361 (7.11) | 836 (8.22) | 468 (9.69) | 354 (7.36) | 822 (8.53) | 7 (2.65) | 7 (2.64) | 14 (2.65) |
| Grade 2 | 40 (0.79) | 21 (0.41) | 61 (0.60) | 39 (0.81) | 20 (0.42) | 59 (0.61) | 1 (0.38) | 1 (0.38) | 2 (0.38) |
| Grade 3 | 3 (0.06) | 2 (0.04) | 5 (0.05) | 2 (0.04) | 2 (0.04) | 4 (0.04) | 1 (0.38) | 0 | 1 (0.19) |
| Grade 4 | 0 | 0 | 0 | 0 | 0 | 0 | 0 | 0 | 0 |
| Pruritus | 30 (0.59) | 44 (0.87) | 74 (0.73) | 29 (0.60) | 40 (0.83) | 69 (0.72) | 1 (0.38) | 4 (1.51) | 5 (0.95) |
| Grade 1 | 28 (0.55) | 43 (0.85) | 71 (0.70) | 27 (0.56) | 39 (0.81) | 66 (0.68) | 1 (0.38) | 4 (1.51) | 5 (0.95) |
| Grade 2 | 1 (0.02) | 1 (0.02) | 2 (0.02) | 1 (0.02) | 1 (0.02) | 2 (0.02) | 0 | 0 | 0 |
| Grade 3 | 1 (0.02) | 0 | 1 (0.01) | 1 (0.02) | 0 | 1 (0.01) | 0 | 0 | 0 |
| Grade 4 | 0 | 0 | 0 | 0 | 0 | 0 | 0 | 0 | 0 |
| Induration | 31 (0.61) | 43 (0.85) | 74 (0.73) | 31 (0.64) | 43 (0.89) | 74 (0.77) | 0 | 0 | 0 |
| Grade 1 | 28 (0.55) | 40 (0.79) | 68 (0.67) | 28 (0.58) | 40 (0.83) | 68 (0.71) | 0 | 0 | 0 |
| Grade 2 | 3 (0.06) | 3 (0.06) | 6 (0.06) | 3 (0.06) | 3 (0.06) | 6 (0.06) | 0 | 0 | 0 |
| Grade 3 | 0 | 0 | 0 | 0 | 0 | 0 | 0 | 0 | 0 |
| Grade 4 | 0 | 0 | 0 | 0 | 0 | 0 | 0 | 0 | 0 |
| Swelling | 20 (0.39) | 29 (0.57) | 49 (0.48) | 20 (0.41) | 29 (0.60) | 49 (0.51) | 0 | 0 | 0 |
| Grade 1 | 18 (0.35) | 25 (0.49) | 43 (0.42) | 18 (0.37) | 25 (0.52) | 43 (0.45) | 0 | 0 | 0 |
| Grade 2 | 2 (0.04) | 4 (0.08) | 6 (0.06) | 2 (0.04) | 4 (0.08) | 6 (0.06) | 0 | 0 | 0 |
| Grade 3 | 0 | 0 | 0 | 0 | 0 | 0 | 0 | 0 | 0 |
| Grade 4 | 0 | 0 | 0 | 0 | 0 | 0 | 0 | 0 | 0 |
| Erythema | 10 (0.20) | 15 (0.30) | 25 (0.25) | 10 (0.21) | 15 (0.31) | 25 (0.26) | 0 | 0 | 0 |
| Grade 1 | 10 (0.20) | 12 (0.24) | 22 (0.22) | 10 (0.21) | 12 (0.25) | 22 (0.23) | 0 | 0 | 0 |
| Grade 2 | 0 | 3 (0.06) | 3 (0.03) | 0 | 3 (0.06) | 3 (0.03) | 0 | 0 | 0 |
| Grade 3 | 0 | 0 | 0 | 0 | 0 | 0 | 0 | 0 | 0 |
| Grade 4 | 0 | 0 | 0 | 0 | 0 | 0 | 0 | 0 | 0 |
| Rash | 6 (0.12) | 5 (0.10) | 11 (0.11) | 6 (0.12) | 5 (0.10) | 11 (0.11) | 0 | 0 | 0 |
| Grade 1 | 6 (0.12) | 4 (0.08) | 10 (0.10) | 6 (0.12) | 4 (0.08) | 10 (0.10) | 0 | 0 | 0 |
| Grade 2 | 0 | 1 (0.02) | 1 (0.01) | 0 | 1 (0.02) | 1 (0.01) | 0 | 0 | 0 |
| Grade 3 | 0 | 0 | 0 | 0 | 0 | 0 | 0 | 0 | 0 |
| Grade 4 | 0 | 0 | 0 | 0 | 0 | 0 | 0 | 0 | 0 |
| Cellulitis | 0 | 2 (0.04) | 2 (0.02) | 0 | 2 (0.04) | 2 (0.02) | 0 | 0 | 0 |
| Grade 1 | 0 | 2 (0.04) | 2 (0.02) | 0 | 2 (0.04) | 2 (0.02) | 0 | 0 | 0 |
| Grade 2 | 0 | 0 | 0 | 0 | 0 | 0 | 0 | 0 | 0 |
| Grade 3 | 0 | 0 | 0 | 0 | 0 | 0 | 0 | 0 | 0 |
| Grade 4 | 0 | 0 | 0 | 0 | 0 | 0 | 0 | 0 | 0 |
| Any Systemic AR | 728 (14.29) | 682 (13.44) | 1410 (13.87) | 705 (14.60) | 662 (13.77) | 1367 (14.18) | 23 (8.71) | 20 (7.55) | 43 (8.13) |
| Grade 1 | 629 (12.35) | 591 (11.65) | 1220 (12.00) | 611 (12.65) | 574 (11.94) | 1185 (12.30) | 18 (6.82) | 17 (6.42) | 35 (6.62) |
| Grade 2 | 184 (3.61) | 158 (3.11) | 342 (3.36) | 181 (3.75) | 155 (3.22) | 336 (3.49) | 3 (1.14) | 3 (1.13) | 6 (1.13) |
| Grade 3 | 33 (0.65) | 24 (0.47) | 57 (0.56) | 32 (0.66) | 23 (0.48) | 55 (0.57) | 1 (0.38) | 1 (0.38) | 2 (0.38) |
| Grade 4 | 3 (0.06) | 0 | 3 (0.03) | 2 (0.04) | 0 | 2 (0.02) | 1 (0.38) | 0 | 1 (0.19) |
| Systemic AR |  |  |  |  |  |  |  |  |  |
| Fever | 321 (6.30) | 279 (5.50) | 600 (5.90) | 306 (6.34) | 270 (5.61) | 576 (5.98) | 15 (5.68) | 9 (3.4) | 24 (4.54) |
| Grade 1 | 203 (3.99) | 202 (3.98) | 405 (3.98) | 193 (4.00) | 195 (4.05) | 388 (4.03) | 10 (3.79) | 7 (2.64) | 17 (3.21) |
| Grade 2 | 88 (1.73) | 60 (1.18) | 148 (1.46) | 85 (1.76) | 59 (1.23) | 144 (1.49) | 3 (1.14) | 1 (0.38) | 4 (0.76) |
| Grade 3 | 28 (0.55) | 17 (0.34) | 45 (0.44) | 27 (0.56) | 16 (0.33) | 43 (0.45) | 1 (0.38) | 1 (0.38) | 2 (0.38) |
| Grade 4 | 3 (0.06) | 0 | 3 (0.03) | 2 (0.04) | 0 | 2 (0.02) | 1 (0.38) | 0 | 1 (0.19) |
| Headache | 268 (5.26) | 248 (4.89) | 516 (5.08) | 265 (5.49) | 240 (4.99) | 505 (5.24) | 3 (1.14) | 8 (3.02) | 11 (2.08) |
| Grade 1 | 209 (4.10) | 195 (3.84) | 404 (3.97) | 207 (4.29) | 188 (3.91) | 395 (4.10) | 2 (0.76) | 7 (2.64) | 9 (1.7) |
| Grade 2 | 59 (1.16) | 54 (1.06) | 113 (1.11) | 58 (1.20) | 53 (1.10) | 111 (1.15) | 1 (0.38) | 1 (0.38) | 2 (0.38) |
| Grade 3 | 0 | 3 (0.06) | 3 (0.03) | 0 | 3 (0.06) | 3 (0.03) | 0 | 0 | 0 |
| Grade 4 | 0 | 0 | 0 | 0 | 0 | 0 | 0 | 0 | 0 |
| Fatigue | 247 (4.85) | 204 (4.02) | 451 (4.44) | 242 (5.01) | 196 (4.08) | 438 (4.54) | 5 (1.89) | 8 (3.02) | 13 (2.46) |
| Grade 1 | 220 (4.32) | 187 (3.69) | 407 (4.00) | 216 (4.47) | 180 (3.74) | 396 (4.11) | 4 (1.52) | 7 (2.64) | 11 (2.08) |
| Grade 2 | 27 (0.53) | 17 (0.34) | 44 (0.43) | 26 (0.54) | 16 (0.33) | 42 (0.44) | 1 (0.38) | 1 (0.38) | 2 (0.38) |
| Grade 3 | 0 | 0 | 0 | 0 | 0 | 0 | 0 | 0 | 0 |
| Grade 4 | 0 | 0 | 0 | 0 | 0 | 0 | 0 | 0 | 0 |
| Myalgia | 165 (3.24) | 141 (2.78) | 306 (3.01) | 161 (3.33) | 140 (2.91) | 301 (3.12) | 4 (1.52) | 1 (0.38) | 5 (0.95) |
| Grade 1 | 138 (2.71) | 113 (2.23) | 251 (2.47) | 134 (2.77) | 112 (2.33) | 246 (2.55) | 4 (1.52) | 1 (0.38) | 5 (0.95) |
| Grade 2 | 27 (0.53) | 26 (0.51) | 53 (0.52) | 27 (0.56) | 26 (0.54) | 53 (0.55) | 0 | 0 | 0 |
| Grade 3 | 1 (0.02) | 2 (0.04) | 3 (0.03) | 1 (0.02) | 2 (0.04) | 3 (0.03) | 0 | 0 | 0 |
| Grade 4 | 0 | 0 | 0 | 0 | 0 | 0 | 0 | 0 | 0 |
| Arthralgia | 96 (1.88) | 81 (1.60) | 177 (1.74) | 95 (1.97) | 79 (1.64) | 174 (1.81) | 1 (0.38) | 2 (0.75) | 3 (0.57) |
| Grade 1 | 87 (1.71) | 75 (1.48) | 162 (1.59) | 86 (1.78) | 73 (1.52) | 159 (1.65) | 1 (0.38) | 2 (0.75) | 3 (0.57) |
| Grade 2 | 9 (0.18) | 6 (0.12) | 15 (0.15) | 9 (0.19) | 6 (0.12) | 15 (0.16) | 0 | 0 | 0 |
| Grade 3 | 0 | 0 | 0 | 0 | 0 | 0 | 0 | 0 | 0 |
| Grade 4 | 0 | 0 | 0 | 0 | 0 | 0 | 0 | 0 | 0 |
| Cough | 88 (1.73) | 86 (1.69) | 174 (1.71) | 87 (1.80) | 82 (1.71) | 169 (1.75) | 1 (0.38) | 4 (1.51) | 5 (0.95) |
| Grade 1 | 72 (1.41) | 74 (1.46) | 146 (1.44) | 71 (1.47) | 70 (1.46) | 141 (1.46) | 1 (0.38) | 4 (1.51) | 5 (0.95) |
| Grade 2 | 16 (0.31) | 12 (0.24) | 28 (0.28) | 16 (0.33) | 12 (0.25) | 28 (0.29) | 0 | 0 | 0 |
| Grade 3 | 0 | 0 | 0 | 0 | 0 | 0 | 0 | 0 | 0 |
| Grade 4 | 0 | 0 | 0 | 0 | 0 | 0 | 0 | 0 | 0 |
| Nausea | 63 (1.24) | 55 (1.08) | 118 (1.16) | 63 (1.30) | 54 (1.12) | 117 (1.21) | 0 | 1 (0.38) | 1 (0.19) |
| Grade 1 | 60 (1.18) | 53 (1.04) | 113 (1.11) | 60 (1.24) | 52 (1.08) | 112 (1.16) | 0 | 1 (0.38) | 1 (0.19) |
| Grade 2 | 3 (0.06) | 2 (0.04) | 5 (0.05) | 3 (0.06) | 2 (0.04) | 5 (0.05) | 0 | 0 | 0 |
| Grade 3 | 0 | 0 | 0 | 0 | 0 | 0 | 0 | 0 | 0 |
| Grade 4 | 0 | 0 | 0 | 0 | 0 | 0 | 0 | 0 | 0 |
| Decreased appetite | 66 (1.30) | 37 (0.73) | 103 (1.01) | 65 (1.35) | 36 (0.75) | 101 (1.05) | 1 (0.38) | 1 (0.38) | 2 (0.38) |
| Grade 1 | 54 (1.06) | 30 (0.59) | 84 (0.83) | 54 (1.12) | 29 (0.60) | 83 (0.86) | 0 | 1 (0.38) | 1 (0.19) |
| Grade 2 | 12 (0.24) | 7 (0.14) | 19 (0.19) | 11 (0.23) | 7 (0.15) | 18 (0.19) | 1 (0.38) | 0 | 1 (0.19) |
| Grade 3 | 0 | 0 | 0 | 0 | 0 | 0 | 0 | 0 | 0 |
| Grade 4 | 0 | 0 | 0 | 0 | 0 | 0 | 0 | 0 | 0 |
| Diarrhoea | 49 (0.96) | 38 (0.75) | 87 (0.86) | 48 (0.99) | 38 (0.79) | 86 (0.89) | 1 (0.38) | 0 | 1 (0.19) |
| Grade 1 | 40 (0.79) | 33 (0.65) | 73 (0.72) | 40 (0.83) | 33 (0.69) | 73 (0.76) | 0 | 0 | 0 |
| Grade 2 | 7 (0.14) | 6 (0.12) | 13 (0.13) | 6 (0.12) | 6 (0.12) | 12 (0.12) | 1 (0.38) | 0 | 1 (0.19) |
| Grade 3 | 2 (0.04) | 1 (0.02) | 3 (0.03) | 2 (0.04) | 1 (0.02) | 3 (0.03) | 0 | 0 | 0 |
| Grade 4 | 0 | 0 | 0 | 0 | 0 | 0 | 0 | 0 | 0 |
| Pruritus-at non-inoculation site | 32 (0.63) | 31 (0.61) | 63 (0.62) | 32 (0.66) | 29 (0.60) | 61 (0.63) | 0 | 2 (0.75) | 2 (0.38) |
| Grade 1 | 29 (0.57) | 30 (0.59) | 59 (0.58) | 29 (0.60) | 28 (0.58) | 57 (0.59) | 0 | 2 (0.75) | 2 (0.38) |
| Grade 2 | 3 (0.06) | 1 (0.02) | 4 (0.04) | 3 (0.06) | 1 (0.02) | 4 (0.04) | 0 | 0 | 0 |
| Grade 3 | 0 | 0 | 0 | 0 | 0 | 0 | 0 | 0 | 0 |
| Grade 4 | 0 | 0 | 0 | 0 | 0 | 0 | 0 | 0 | 0 |
| Dyspnoea | 27 (0.53) | 26 (0.51) | 53 (0.52) | 27 (0.56) | 26 (0.54) | 53 (0.55) | 0 | 0 | 0 |
| Grade 1 | 18 (0.35) | 16 (0.32) | 34 (0.33) | 18 (0.37) | 16 (0.33) | 34 (0.35) | 0 | 0 | 0 |
| Grade 2 | 7 (0.14) | 9 (0.18) | 16 (0.16) | 7 (0.14) | 9 (0.19) | 16 (0.17) | 0 | 0 | 0 |
| Grade 3 | 2 (0.04) | 1 (0.02) | 3 (0.03) | 2 (0.04) | 1 (0.02) | 3 (0.03) | 0 | 0 | 0 |
| Grade 4 | 0 | 0 | 0 | 0 | 0 | 0 | 0 | 0 | 0 |
| Dysphagia | 24 (0.47) | 22 (0.43) | 46 (0.45) | 24 (0.50) | 21 (0.44) | 45 (0.47) | 0 | 1 (0.38) | 1 (0.19) |
| Grade 1 | 23 (0.45) | 20 (0.39) | 43 (0.42) | 23 (0.48) | 19 (0.40) | 42 (0.44) | 0 | 1 (0.38) | 1 (0.19) |
| Grade 2 | 1 (0.02) | 2 (0.04) | 3 (0.03) | 1 (0.02) | 2 (0.04) | 3 (0.03) | 0 | 0 | 0 |
| Grade 3 | 0 | 0 | 0 | 0 | 0 | 0 | 0 | 0 | 0 |
| Grade 4 | 0 | 0 | 0 | 0 | 0 | 0 | 0 | 0 | 0 |
| Constipation | 21 (0.41) | 23 (0.45) | 44 (0.43) | 21 (0.43) | 21 (0.44) | 42 (0.44) | 0 | 2 (0.75) | 2 (0.38) |
| Grade 1 | 20 (0.39) | 22 (0.43) | 42 (0.41) | 20 (0.41) | 20 (0.42) | 40 (0.42) | 0 | 2 (0.75) | 2 (0.38) |
| Grade 2 | 1 (0.02) | 1 (0.02) | 2 (0.02) | 1 (0.02) | 1 (0.02) | 2 (0.02) | 0 | 0 | 0 |
| Grade 3 | 0 | 0 | 0 | 0 | 0 | 0 | 0 | 0 | 0 |
| Grade 4 | 0 | 0 | 0 | 0 | 0 | 0 | 0 | 0 | 0 |
| Vomiting | 19 (0.37) | 16 (0.32) | 35 (0.34) | 19 (0.39) | 16 (0.33) | 35 (0.36) | 0 | 0 | 0 |
| Grade 1 | 16 (0.31) | 11 (0.22) | 27 (0.27) | 16 (0.33) | 11 (0.23) | 27 (0.28) | 0 | 0 | 0 |
| Grade 2 | 3 (0.06) | 5 (0.10) | 8 (0.08) | 3 (0.06) | 5 (0.10) | 8 (0.08) | 0 | 0 | 0 |
| Grade 3 | 0 | 0 | 0 | 0 | 0 | 0 | 0 | 0 | 0 |
| Grade 4 | 0 | 0 | 0 | 0 | 0 | 0 | 0 | 0 | 0 |
| Mucocutaneous disorder | 15 (0.29) | 13 (0.26) | 28 (0.28) | 15 (0.31) | 12 (0.25) | 27 (0.28) | 0 | 1 (0.38) | 1 (0.19) |
| Grade 1 | 13 (0.26) | 9 (0.18) | 22 (0.22) | 13 (0.27) | 8 (0.17) | 21 (0.22) | 0 | 1 (0.38) | 1 (0.19) |
| Grade 2 | 2 (0.04) | 3 (0.06) | 5 (0.05) | 2 (0.04) | 3 (0.06) | 5 (0.05) | 0 | 0 | 0 |
| Grade 3 | 0 | 1 (0.02) | 1 (0.01) | 0 | 1 (0.02) | 1 (0.01) | 0 | 0 | 0 |
| Grade 4 | 0 | 0 | 0 | 0 | 0 | 0 | 0 | 0 | 0 |
| Hypersensitivity | 7 (0.14) | 7 (0.14) | 14 (0.14) | 7 (0.14) | 7 (0.15) | 14 (0.15) | 0 | 0 | 0 |
| Grade 1 | 7 (0.14) | 5 (0.10) | 12 (0.12) | 7 (0.14) | 5 (0.10) | 12 (0.12) | 0 | 0 | 0 |
| Grade 2 | 0 | 2 (0.04) | 2 (0.02) | 0 | 2 (0.04) | 2 (0.02) | 0 | 0 | 0 |
| Grade 3 | 0 | 0 | 0 | 0 | 0 | 0 | 0 | 0 | 0 |
| Grade 4 | 0 | 0 | 0 | 0 | 0 | 0 | 0 | 0 | 0 |

1. n=Number of exposed participants who submitted any data for the event; percentages are based on the number of exposed participants who submitted any data for the event in the solicited safety set. N=Number of exposed participants who have been collected diary cards.
2. Adverse reactions are defined as any harmful or unintended reactions possibly attributed to the use of investigational vaccine during the clinical trial. If the causal relationship between investigational vaccine and AE implies at least a reasonable possibility, i.e., relevance cannot be excluded.
3. Any Local AR including pain, pruritus, redness, swelling, skin rash, induration and cellulitis;
4. Any Systemic AR including fever, diarrhea, constipation, dysphagia, anorexia, vomiting, nausea, myalgia, arthralgia, headache, cough, dyspnea, non-inoculation site pruritus, abnormal cutaneous mucosa (skin rash, pruritus, etc.), fatigue and acute allergic reactions.

**Table S5.** Unsolicited Adverse Events 28 Days after Vaccination, Safety Set

| Unsolicited Adverse Event n (%) | Overall Safety Set | | | ≥18-<60 years | | | ≥60 years | | |
| --- | --- | --- | --- | --- | --- | --- | --- | --- | --- |
|  | Placebo Group | V-01 Group | Total | Placebo Group | V-01 Group | Total | Placebo Group | V-01 Group | Total |
|  | (N=5110) | (N=5108) | (N=10218) | (N=4845) | (N=4841) | (N=9686) | (N=265) | (N=267) | (N=532) |
| Regardless of relationship to study vaccination |  |  |  |  |  |  |  |  |  |
| All | 177 (3.46) | 174 (3.41) | 351 (3.44) | 173 (3.57) | 166 (3.43) | 339 (3.50) | 4 (1.51) | 8 (3.00) | 12 (2.26) |
| Fatal | 0 | 0 | 0 | 0 | 0 | 0 | 0 | 0 | 0 |
| Leading to discontinuation from study vaccine | 0 | 0 | 0 | 0 | 0 | 0 | 0 | 0 | 0 |
| Severe (≥Grade 3) | 6 (0.12) | 3 (0.06) | 9 (0.09) | 4 (0.08) | 3 (0.06) | 7 (0.07) | 2 (0.75) | 0 | 2 (0.38) |
|  |  |  |  |  |  |  |  |  |  |
| Related to study vaccination |  |  |  |  |  |  |  |  |  |
| All | 58 (1.14) | 45 (0.88) | 103 (1.01) | 58 (1.20) | 45 (0.93) | 103 (1.06) | 0 | 0 | 0 |
| Fatal | 0 | 0 | 0 | 0 | 0 | 0 | 0 | 0 | 0 |
| Leading to discontinuation from study vaccine | 0 | 0 | 0 | 0 | 0 | 0 | 0 | 0 | 0 |
| Severe (≥Grade 3) | 0 | 2 (0.04) | 2 (0.02) | 0 | 2 (0.04) | 2 (0.02) | 0 | 0 | 0 |

n=Number of exposed participants who submitted any data for the event; percentages are based on the number of exposed participants who submitted any data for the event in the Overall Safety Set. N=Number of exposed participants in the Overall Safety Set.

**Figure S1**. Efficacy of heterologous boost with V-01 vaccine preventing COVID-19 in subgroup according to PP analysis


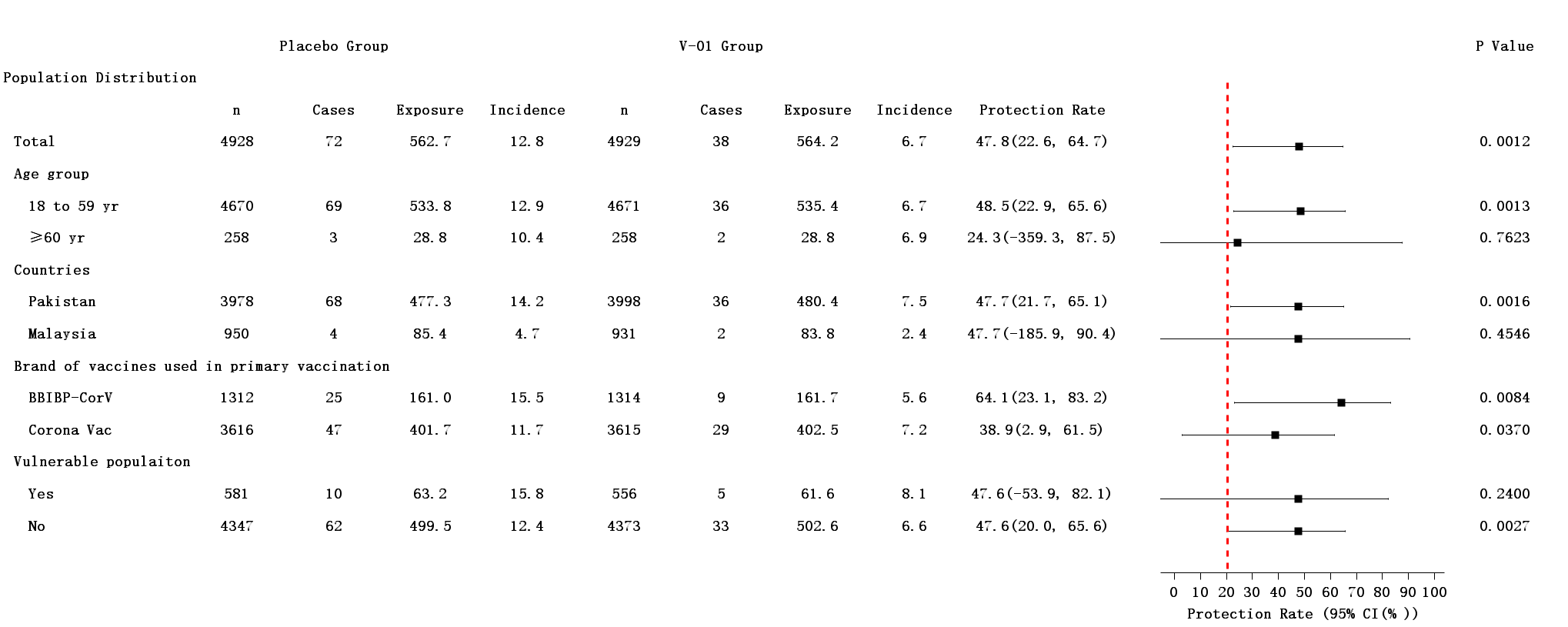


**Supplementary document 1**. Ethical approvals from all study centers

Totally 15 centers were included in this trial. Of these, 10 centers coded as 6001 to 6010 located in Malaysia, and 5 centers coded as 9201, 9202/9203, 9203/9204, 9205 and 9208 located in Pakistan. The identification numbers (ID No.) of ethical approval were listed as following.

1. Malaysia
2. Center 6001、6002、6003、6004、6007、6008、6009、6010 locate in same region, and share one ethical committee. The ID No. is NMRR-21-1565-61053 (ISR);
3. Center 60025 and 6006 locate in another region, the ID No. is 011/2021/IND/FR;
4. Pakistan
5. Center 9201 and 9208 had approval letter without ID No.;
6. Center 9202/9203: IRB-2077/DUHS;
7. Center 9203/9204: IRB #:162-61;
8. Center 9205: IRB/01-8/21/AVC.
